# Risk factors for chronic kidney disease of non-traditional causes: a systematic review

**DOI:** 10.26633/RPSP.2019.35

**Published:** 2019-03-14

**Authors:** Evelina Chapman, Michelle M. Haby, Eduardo Illanes, Julian Sanchez-Viamonte, Vanessa Elias, Ludovic Reveiz

**Affiliations:** 1 Campus Universitário Darcy Ribeiro Campus Universitário Darcy Ribeiro Oswaldo Cruz Foundation (Fiocruz) Brasilia Brazil Oswaldo Cruz Foundation (Fiocruz), Campus Universitário Darcy Ribeiro, Brasilia, Brazil.; 2 Universidad de Sonora Universidad de Sonora Department of Chemical and Biological Sciences HermosilloSonora Mexico Department of Chemical and Biological Sciences, Universidad de Sonora, Hermosillo, Sonora, Mexico.; 3 Universidad Mayor, a Ringgold standard institution Universidad Mayor, a Ringgold standard institution School of Psychology Santiago Chile School of Psychology, Universidad Mayor, a Ringgold standard institution, Santiago, Chile.; 4 Facultad de Ciencias Médicas de la Universidad Nacional de La Plata Facultad de Ciencias Médicas de la Universidad Nacional de La Plata Escuela Universitaria de Recursos Humanos del Equipo de Salud Informática en Ciencias de la Salud Buenos Aires Argentina Informática en Ciencias de la Salud, Escuela Universitaria de Recursos Humanos del Equipo de Salud, Facultad de Ciencias Médicas de la Universidad Nacional de La Plata, Buenos Aires, Argentina.; 5 Pan American Health Organization/World Health Organization Pan American Health Organization/World Health Organization Department of Evidence and Intelligence for Action in Health WashingtonDC United States of America Department of Evidence and Intelligence for Action in Health, Pan American Health Organization/World Health Organization, Washington, DC, United States of America.

**Keywords:** Renal insufficiency, chronic, agricultural workers’ diseases, agrochemicals, heat exhaustion, meta-analysis, Insuficiencia renal crónica, enfermedades de los trabajadores agrícolas, agroquímicos, agotamiento por calor, metaanálisis, Insuficiência renal crônica, doenças dos trabalhadores agrícolas, agroquímicos, exaustão por calor, metanálise

## Abstract

**Objectives.:**

To evaluate the potential associations between chronic kidney disease of uncertain or non-traditional etiology (CKDnT) and agrochemicals, heat stress, heavy metals, and other factors identified in the literature in any region of the world and at any time.

**Methods.:**

This was a systematic review of the most frequent exposures suspected to be possible causes of CKDnT. A search was conducted of PubMed, LILACS, World Wide Science electronic databases, among other sources. Only medium- and high-quality studies were included. The synthesis of evidence included a narrative synthesis, meta-analysis, and meta-regression.

**Results.:**

Four systematic reviews and 61 primary studies were included. Results of the meta-analysis suggest that exposure to agrochemicals and working in agriculture increase the risk of CKDnT, but this only reached significance for working in agriculture. When cross-sectional studies were excluded, agrochemical exposure became significant. However, there is substantial heterogeneity in the effect sizes.

**Conclusions.:**

Based on the existing evidence and the precautionary principle, it is important to implement preventive measures to mitigate the damage caused by CKDnT to both agricultural workers and their communities (i.e., improvement of working conditions, cautious management of agrochemicals, etc.). More high-quality research is needed to measure impact and to build the evidence base.

## RESUMEN

**Objetivos.:**

Evaluar las posibles asociaciones de la enfermedad renal crónica de etiología incierta o no tradicional (ERCnT) con los agroquímicos, el estrés por calor, los metales pesados y otros factores señalados en la bibliografía de cualquier región del mundo y en cualquier período.

**Métodos.:**

La presente es una revisión sistemática de las exposiciones más frecuentes que se sospecha podrían ser posibles causas de la ERCnT. Se realizó una búsqueda en las bases de datos electrónicas de PubMed, LILACS y World Wide Science, entre otras fuentes. Se incluyeron solamente estudios de calidad media y alta. La síntesis de la evidencias incluyó síntesis narrativa, metanálisis y metarregresión.

**Resultados.:**

Se incluyeron cuatro revisiones sistemáticas y 61 estudios primarios. Los resultados del metaanálisis sugieren que la exposición a agroquímicos y el trabajo agrícola aumentan el riesgo de ERCnT, pero esto solo alcanzó un nivel significativo con respecto al trabajo agrícola. Al excluir los estudios transversales, la exposición a agroquímicos se tornó significativa. Sin embargo, existe una heterogeneidad sustancial en las magnitudes del efecto.

**Conclusiones.:**

Con base en las pruebas científicas existentes y en el principio de precaución, es importante aplicar medidas preventivas para mitigar el daño ocasionado por la ERCnT tanto para los agricultores como para sus comunidades (es decir, mejoras en las condiciones de trabajo, uso prudente de agroquímicos, etc.). Es necesario aumentar la investigación de alta calidad para medir el impacto y ampliar la base de pruebas científicas.

## RESUMO

**Objetivos.:**

Avaliar as associações em potencial entre doença renal crônica de etiologia incerta ou não tradicional e produtos agroquímicos, estresse térmico, metais pesados e outros fatores identificados na literatura em qualquer região do globo ou espaço de tempo.

**Métodos.:**

Trata-se de uma revisão sistemática dos tipos mais comuns de exposição suspeitas de serem possíveis causas de doença renal crônica de etiologia incerta ou não tradicional. Foram pesquisadas as bases de dados eletrônicas PubMed, LILACS, World Wide Science, entre outras fontes. Somente foram incluídos estudos de qualidade intermediária e alta. Síntese narrativa, meta-análise e metarregressão foram usadas para sumarizar as evidências.

**Resultados.:**

Este estudo compreendeu quatro estudos de revisão sistemática e 61 estudos primários. Os resultados da meta-análise indicaram que a exposição a produtos agroquímicos e o trabalho na agricultura aumentam o risco de doença renal crônica de etiologia incerta ou não tradicional, com risco significativo apenas para o trabalho na agricultura. Após a exclusão dos estudos transversais, a exposição a produtos agroquímicos também representou um risco significativo. Porém, observou-se considerável heterogeneidade nos tamanhos do efeito.

**Conclusões.:**

Diante das evidências existentes e com base no princípio da precaução, é importante implementar medidas preventivas para atenuar os danos causados pela doença renal crônica de etiologia incerta ou não tradicional aos trabalhadores e às comunidades agrícolas, como melhoria das condições de trabalho e manipulação cautelosa dos produtos agroquímicos, entre outros. Faz-se necessário realizar outras pesquisas de alta qualidade para avaliar o impacto e aumentar a base de evidências.

During the last four decades, a severe form of renal failure has increased among individuals living in socially-vulnerable farming communities of Central America and Asia. In particular, El Salvador and Nicaragua have seen important increases in patients with chronic kidney disease (CKD) and CKD mortality (1). Parts of the Region of the Americas have experienced up to four times the global CKD mortality rate (1).

Chronic kidney disease of uncertain or non-traditional etiology (CKDnT) is a severe form of the disease characterized by progressive renal failure, often diagnosed at a very advanced stage due to the absence of early symptoms; it requires replacement therapy for survival (2). Its etiology is not linked to diabetes, hypertension, glomerulopathies, or other known causes of renal diseases. The clinical, biochemical, and histological characterizations of CKDnT in South Asia, particularly in Sri Lanka, and Central America are very similar (1 – 3).

The underlying etiologies for CKDnT have not been clearly identified in most regions, but two predominant groups of suspected risk factors have been studied (4). The first group is related to repeated and prolonged exposure to potential toxins, including pesticides and heavy metals, in drinking water and in agricultural communities (1). The second is related to heat stress with repeated episodes of dehydration. A recent systematic review of pesticides and CKDnT found scarce evidence for an association with regional CKDnT epidemics, but the role of agrochemicals could not be conclusively discarded based on the evidence available at the time of the review (5).

While there is a general consensus that the patterns of CKDnT are similar in Central America and South Asia, no meta-analysis of all the important risk factors without country limitations had been conducted to date. The objective of this systematic review was to evaluate the potential associations between CKDnT and agrochemicals, heat-stress dehydration, heavy metals, hard water, and other factors described in the literature in any region of the world and at any time.

## MATERIALS AND METHODS

A systematic review and meta-analysis (6 – 8) were conducted according to the study’s previously registered protocol (9). The search included systematic reviews, experimental studies, and observational studies of medium or high quality among any populations with CKDnT. Any exposure implicated in the literature was included as a possible cause of CKDnT, as was any comparison.

The primary outcome was CKDnT defined as estimated glomerular filtration rate (eGFR) < 60 mL/min/1.73m2 body surface area and/or non-nephrotic proteinuria and/or urinary sediment abnormalities as markers of kidney damage and/or renal tubular disorders and exclusion of traditional causes of CKD (2). Acute kidney injury as a proxy outcome measure for studies of heat stress-dehydration was also included.

Studies included were published in English, Spanish, or Portuguese. There was no restriction on the year of publication for systematic reviews; however, for primary studies, only those published after the search dates of the most recent systematic review were included. Searches were conducted of PubMed Central (U.S. National Library of Medicine, Bethesda, Maryland, United States), LILACS (Latin American and Caribbean Center on Health Sciences Information, PAHO/WHO, São Paulo, Brazil), World Wide Science electronic databases (Office of Scientific and Technical Information, U.S. Department of Energy, Oak Ridge, Tennessee, United States), GreenFILE (EBSCO, Ipswich, Massachusetts, United States), AGRIS (Food and Agriculture Organization of the United Nations, Rome, Italy), and PLAGSALUD (MASICA/OPS) using a combination of subject headings and text words for kidney disease and the potential exposures, developed from previous systematic reviews (4, 5). Examples of search strategies are available in the Supplementary Materials. The last search was conducted in August 2017. References and grey literature were also searched, and experts were also consulted. Search results were screened, according to the selection criteria, by one review author; while full texts were retrieved and reviewed against the inclusion criteria by two reviewers. Disagreements were resolved by discussion and consensus.

Data were extracted by one reviewer into Microsoft Word™ tables (Microsoft Corp., Redmond, Washington, United States), and then checked by a second reviewer. Data extracted included: exposure, study design, sample size, population, country of study, definition of CKDnT, results, funding source, and research gaps. Primary studies already included in systematic reviews did not undergo detailed data extraction or quality assessment, except where required for the meta-analysis. Exposure was classified as: agrochemicals, heavy metals, hard water, heat stress-dehydration, working in agriculture, and other exposures. CKDnT outcomes were classified as: 1 – eGFR with or without non-nephrotic albuminuria; 2 – non-nephrotic albuminuria/proteinuria; 3 – end-stage renal disease (ESRD); and 4 – mortality.

The quality of included studies was assessed by two reviewers. Disagreements were resolved through discussion and consensus. Low quality studies were excluded from the review. For systematic reviews, Assessing the Methodological Quality of Systematic Reviews (AMSTAR) was used (10); scores from 8 – 11 were considered to be high quality; from 4 – 7 of medium quality; and 0 – 3 of low quality.

The Newcastle-Ottawa Quality Assessment Scale was used for cohort studies and case control studies. Scores from 7 – 9 were considered to be of high quality; from 4 – 6 of medium quality; and from 0 – 3 of low quality. Cross-sectional studies were assessed using the adapted RTI Item Bank instrument proposed by the Agency for Healthcare Research and Quality (11).

The synthesis of evidence was based on both a qualitative synthesis and meta-analysis, when possible. This review reports both the effect of the exposure on CKDnT and whether study showed a dose-response relationship between exposure and outcome. Gaps in the research evidence were also identified.

### Meta-analysis and meta-regression

Meta-analysis was used to determine the odds ratio (OR) for each of the main exposures: agrochemicals, heat stress-dehydration, heavy metals (including the subcategories arsenic, cadmium, lead, and mercury), hard water, and working in agriculture. ORs were adjusted for other risk factors whenever available, and were chosen because they were the most commonly used statistic in the included primary studies. MetaXL was used to conduct the meta-analysis using the inverse variance heterogeneity method (12, 13). Heterogeneity was assessed using Cochran’s Q and I^2^ statistics. Doi plots and the LFK index were used to evaluate the presence of small-study effects, where asymmetry can indicate publication or other biases (14).

Given the substantial heterogeneity, a meta-regression was conducted to determine the factors that impact on the effect sizes (6). MetaXL was used to prepare the data for further analysis in Stata^®^/MP14 (StataCorp LP, College Station, Texas, United States) using the study weights generated (12, 13). Effect sizes (lnOR) were transformed. The primary aim of the analysis was to optimize the amount of variance explained using a multivariate linear regression model. A multivariate model was created by adding one factor at a time in order of the amount of variability it explained (R-squared) in univariate analyses. Models were compared using the adjusted R-squared statistic. If the last factor introduced into the model did not increase the adjusted R-squared, it was removed from the model before adding the next factor. The significance of a group of variables was tested using a Wald test.

### CKDnT causality considerations

The various exposures addressed in the literature were compared against the nine considerations for causality of Bradford Hill (15, 16).

## RESULTS

**FIGURE 1:**
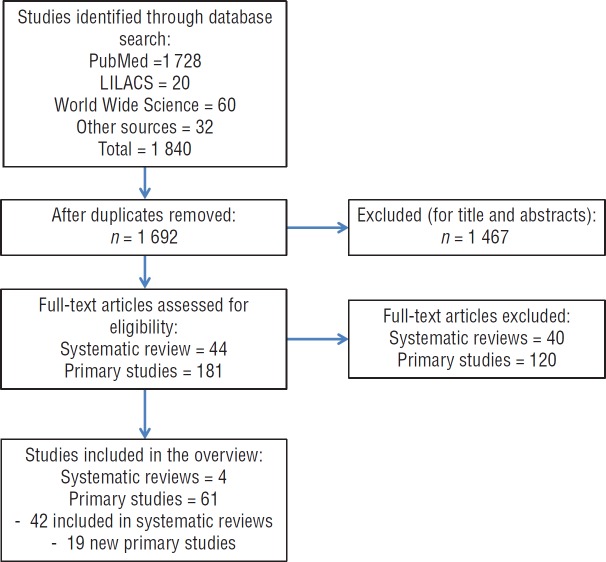
Study selection flowchart for systematic review of risk factors for chronic kidney disease of non-traditional causes

***Source:*** Prepared by the authors from study data.

In all, 4 systematic reviews (4, 5, 17, 18) and 61 primary studies were included (19 – 79), 19 of which came from the search for primary studies (61 – 79). The selection process for studies and the numbers at each stage are shown in Figure 1. Reasons for exclusion of systematic reviews and primary studies at full text stage, as well as characteristics of included studies and their quality assessment scores are compiled in the Supplementary Materials.

### Meta-analysis and meta-regression

**FIGURE 2:**
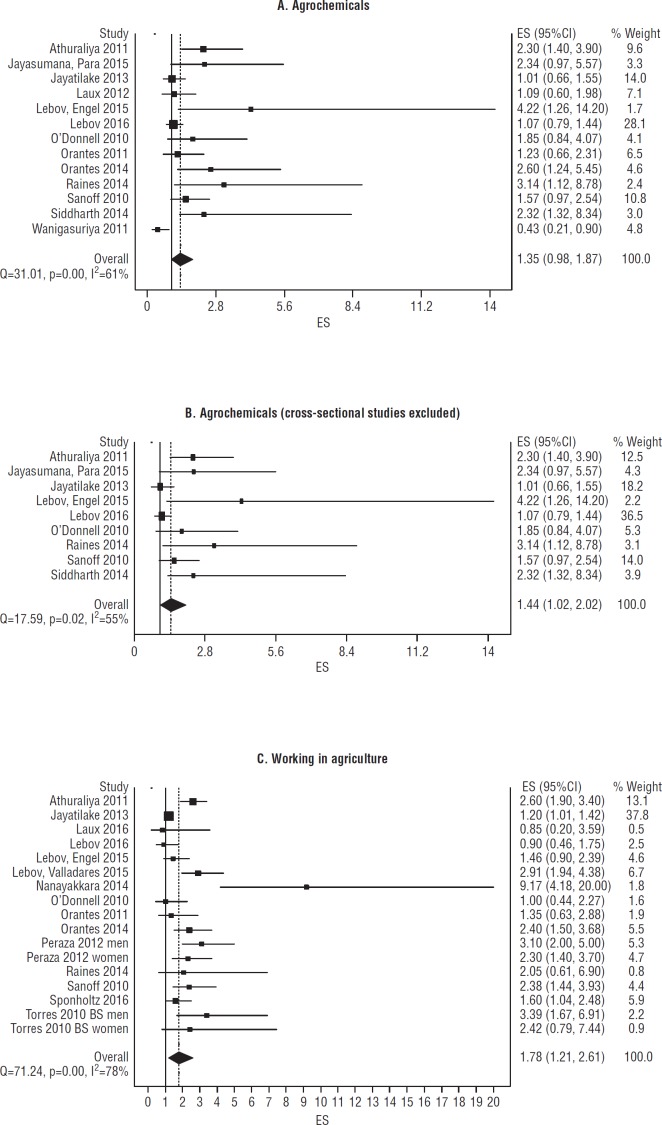
Forest plots showing the odds ratio for the effect of (A) agrochemicals (all studies); (B) agrochemicals (cross-sectional studies excluded); and (C) working in agriculture on any CKDnT outcome. The summary odds ratio was generated using the inverse variance heterogeneity model.

***Source:*** Prepared by the authors from study data.

**FIGURE 3:**
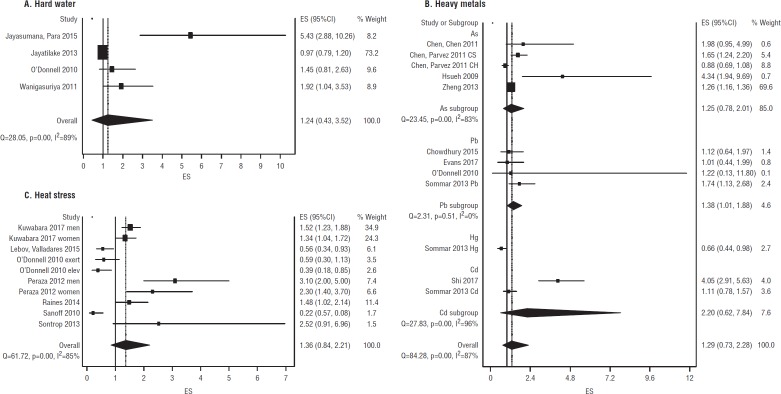
Forest plots showing the odds ratio for the effect of (A) hard water; (B) heavy metals; and (C) heat stress on any CKDnT outcome. The summary odds ratio was generated using the inverse variance heterogeneity model.

***Source:*** Prepared by the authors from study data.

ORs (or equivalent) were available for 56 exposure-control comparisons from 29 studies. As seen in Figures 2 and 3, all exposures had combined ORs > 1, but this was only consistent for exposure to agrochemicals and working in agriculture, which was also statistically significant. Furthermore, analyses showed substantial heterogeneity, with I^2^ values from 61% – 89% (7). The Doi plots showed major asymmetry for agrochemicals, hard water, and heat stress-dehydration; in contrast, Doi plots also showed only minor asymmetry for working in agriculture and heavy metals. Further details are available in the Supplementary Materials (S5).

The results and interpretation of the meta-regression showed that only exposure and outcome were significant in the multivariate model, but region of study, population of study, and study design were included because they helped to explain the variation in the effect sizes. Together, these variables explained 59% of the between-study variance in the effect sizes (R^2^ = 0.587), compared to 9.1% for exposure alone (R^2^ = 0.091).

### Agrochemicals / working in agriculture

Two systematic reviews (4, 5) and 25 primary studies (27 articles; 20, 26, 27, 30 – 34, 38, 40 – 42, 44, 45, 48, 50, 51, 57 – 59, 63, 66, 67, 75, 77) contributed data on exposure to agrochemicals and/or working in agriculture (see the Supplementary Materials for more details). The majority of studies were conducted in Central America (*n* = 14) or South Asia (*n* = 7).

#### Agrochemicals.

Exposure to agrochemicals increased the risk of CKDnT (OR 1.35; 95%CI = 0.98 - 1.87; 13 studies), but the result was not significant and there was substantial heterogeneity (I^2^ = 61%), as shown in Figure 2A. When the cross-sectional studies were excluded from the analysis (Figure 2B), the OR increased and became significant (OR 1.44; 95%CI = 1.02 – 2.02; 9 studies; I^2^=55%). Of 3 studies not included in the meta-analysis (see the Supplementary Materials S6.1), one showed exposure to agrochemicals to be a risk for CKDnT (31, 32), though the result was not significant, and the other showed agrochemicals to be protective (34). The third study only had results for specific organochlorine pesticides (63).

#### Working in agriculture.

Working in agriculture was a significant risk for CKDnT (OR 1.78; 95%CI = 1.21 – 1.61; 17 comparisons from 15 studies; I^2^=78%), as shown in Figure 2C. Of the three studies not included in the meta-analysis, two were positive and statistically significant (31, 32, 77) and the other (59) did not provide sufficient data to know the direction of the effect (see the Supplementary Materials S6.1).

### Heavy metals / hard water

Three systematic reviews (4, 17, 18) and 27 primary studies (19, 21 – 29, 35 – 40, 43, 46, 47, 53, 56, 58, 60 – 62, 73, 76) contributed data on heavy metals and/or hard water. One of the included primary studies contained both cross-sectional and cohort components, with each showing different results (22); and one cross-sectional study also included a case-control component for exposure to heavy metals (27), making a total number of 29 primary studies (see the Supplementary Materials). The majority of studies were conducted in South Asia or non-endemic countries and in adults.

#### Heavy metals.

Exposure to heavy metals increased the risk of CKDnT (OR 1.29; 95%CI = 0.73 – 2.28), but the result was not significant and had substantial heterogeneity (I^2^ = 87%), as shown in Figure 3B. However, there were four clear subgroups for analysis of results, i.e., arsenic, cadmium, mercury, and lead. This analysis showed that only lead was a significant risk—combined OR 1.38 (95%CI = 1.01 – 1.88; 4 studies). Furthermore, there was no significant heterogeneity in the lead results (I^2^ = 0%). As shown in Figure 3B, the only study of mercury that contributed data to the meta-analysis showed a significant protective effect (OR 0.66; 95%CI = 0.44 – 0.98); however, only 12 of the 26 primary studies contributed data since the others did not have ORs or equivalents.

When the results from the qualitative analysis were considered (26 studies), the results were less clear (see the Supplementary Materials S6.2). For lead, 9 studies reported results, 4 of which showed exposure to be a risk for CKDnT (all of which were included in the meta-analysis); 3 showed it to be protective; and 2 did not provide sufficient data to know the direction of the effect. Moreover, 3 studies showed a positive dose-response relationship and 3 found no trend. For arsenic, 5 of the 13 studies showed the exposure to be a risk, while 6 found it to be protective, 5 found a positive and statistically significant dose-response relationship, and 2 found a negative dose-response relationship. For cadmium, 7 of the 14 studies found exposure to be a risk and 3 found it to be protective. Further, 6 of the 14 studies found a positive dose-response relationship rather than a negative one (*n* = 1). For mercury, the 2 included studies showed a protective effect and 1 of these also found a negative dose-response relationship.

#### Hard water.

Consumption of hard water increased the risk of CKDnT (OR 1.24; 95%CI = 0.43 – 3.52; 4 studies), but the result was not significant and had substantial heterogeneity (I^2^=89%), as shown in Figure 3A. While 3 of the 4 studies showed hard water to be a risk and 1 also found a positive dose-response effect, a large study showed a small protective effect of hard water (see the Supplementary Materials S6.2). While the 4 included studies were classified as high quality, none were cohort studies.

### Heat stress-dehydration (adverse conditions at work)

One systematic review of medium quality (4) and 8 primary studies (40, 44, 45, 48, 54, 65, 67, 68) contributed data on heat stress-dehydration. Five of the primary studies were conducted in Central America and three in non-declared endemic countries (see the Supplementary Materials). All studies used eGFR as the outcome measure with the exception of Moyce (68), which measured acute kidney injury. A wide variety of exposure measures were used as proxy measures of heat stress-dehydration (see the Supplementary Materials S6.3) and the cohort studies all had very short follow-up times.

**TABLE 1:** Characteristics and results of studies that measured the effect of other exposures on CKDnT

**Study author & year**		**Country**	**Region**^a^	**Study design**^b^	**Quality**	**Sample size, *n***	**Population**^c^	**Proportion male, %**	**Outcome**^d^	**Effect**^e^	**Dose-response**^e^
**Alcohol**											
	Jayatilake 2013 (27)	Sri Lanka	1	CC	high	4 462	general	43.7	2	(+) NS	
	Lebov, Valladares 2015 (67)	Nicaragua	2	CS	high	2 275	general	41.8	1	(+) SS	
	O’Donnell 2011 (40)	Nicaragua	2	CC	high	319	general	50.5	1	(+) NS	
	Raines 2014 (45)	Nicaragua	2	CC	high	117	general	70.1	1	(+) NS	
	Sanoff 2010 (48)	Nicaragua	2	CC	high	334	general	100.0	1	(+) SS	
	Stanifer 2015 (55)	Tanzania	3	CS	high	481	general	25.6	1	(+) NS	
	Wesseling 2016a (59)	Nicaragua	2	CS	medium	194	occupational	100.0	1	(+) NS	
	Xing 2015 (78)	China	3	CS	medium	241	hospital	62.0	1	(+) SS	
	Yang 2015 (79)	Taiwan	3	CH	medium	88	hospital	38.2	1	(+) NS	
**Smoking**											
	Jayatilake 2013 (27)	Sri Lanka	1	CC	high	4 462	general	43.7	2	(+) NS	
	Lebov, Valladares 2015 (67)	Nicaragua	2	CS	high	2 275	general	41.8	1	(-) NS	
	Nanayakkara 2014 (38)	Sri Lanka	1	CC	high	597	general	100.0	1	(+) NS	
	O’Donnell 2011 (40)	Nicaragua	2	CC	high	319	general	50.5	1	(+) NS	
	Peraza 2012 (44) (crude OR)	El Salvador	2	CS	high	256	occupational	100.0	1	(+) NS	
	Raines 2014 (45)	Nicaragua	2	CC	high	117	general	70.1	1	(+) NS	
	Wanigasuriya 2011 (58)	Sri Lanka	1	CC	high	886	general	52.0	2	(+) SS	
	Wesseling 2016a (59)	Nicaragua	2	CS	medium	194	occupational	100.0	1	(+) NS	
	Yang 2015 (79)	Taiwan	3	CH	medium	88	hospital	38.2	1	(+) NS	
**Diet**											
	Jelaković 2015 (64)	Croatia	4	CS	medium	2 161	general	37.5	1	(+) SS	
	Raines 2014 (45)	Nicaragua	2	CC	high	117	general	70.1	1	(+) SS	
	Shi 2017 (73)	China	3	CH	high	8 429	general	47.0	1	(+) SS	(+) SS
	Siriwardhana 2014 & 2015 (52, 74)	Sri Lanka	1	CC	medium	200	hospital	59.0	1	( ) SS	
**Genetics**											
	Athuraliya 2011 (20)	Sri Lanka	1	CS	high	6 153	general	47.0	1	(+) SS	
	Nanayakkara 2014 (38)	Sri Lanka	1	CC	high	597	general	100.0	1	(+) SS	
	Nanayakkara 2015 (69)	Sri Lanka	1	CC	medium	504	occupational	65.0	1	(+) SS	
	Orantes 2011 (41)	El Salvador	2	CS	medium	775	occupational	44.3	1	(+) SS	
	Sanoff 2010 (48)	Nicaragua	2	CC	high	334	general	100.0	1	(+) NS	
	Sayanthooran 2016 (72)	Sri Lanka	1	CC	medium	43	occupational	86.0	1	(+) SS	
	Sayanthooran 2017 (71)	Sri Lanka	1	CC	medium	60	occupational	85.0	1	(+) SS	
	Siddarth 2013 (49)	India	1	CC	medium	668	hospital	50.0	1	(+) SS	
**Communicable diseases**											
	Riefkhol 2017 (70)	Nicaragua	2	CH	medium	489	occupational	100.0	1	(+) SS	(+) SS
	Yang 2015 (79)	Taiwan	3	CH	medium	88	hospital	38.2	1	(+) SS	(+) SS
**Snakebite**											
	Nanayakkara 2014 (38)	Sri Lanka	1	CC	high	597	general	100.0	1	(+) SS	
**Tobacco chewing**											
	Nanayakkara 2014 (38)	Sri Lanka	1	CC	high	597	general	100.0	1	(+) SS	

a Regions: South Asia = 1, Central America = 2, and Non-endemic = 3, Balkan States = 4

b Study design: CC = case-control, CH = cohort, CS = cross-sectional

c Population: hospital = hospital-based

d eGFR (with or without non-nephrotic albuminuria) = 1, non-nephrotic albuminuria/proteinuria = 2, end-stage renal disease = 3, mortality = 4

e (+) = positive; (-) = negative; ( ) OR not reported (just 95% CI and *P* value); SS = statistically significant effect (*P* < 0.05); NS = not significant.

***Source:*** Prepared by the authors from study data.

Exposure to heat stress-dehydration increased the risk of CKDnT (OR 1.36; 95%CI = 0.84 – 2.21) according to 7 studies with 10 comparisons (40, 44, 45, 48, 54, 65, 67)—results not significant and with substantial heterogeneity (I^2^ = 85%), as shown in Figure 3C. Furthermore, 4 of the 10 comparisons showed a protective effect against CKDnT. The only study (68) not included in the meta-analysis used a direct measure of heat stress-dehydration, but measured acute kidney injury instead of CKDnT (see the Supplementary Materials S6.3). Three studies found a positive dose-response relationship between heat stress-dehydration and CKDnT (44, 54, 65), 2 of which were statistically significant (44, 65) and 2 found a statistically significant negative dose-response relationship (48, 67).

### Other exposures

One systematic review of medium quality (4) and 23 primary studies contributed data on other exposures (20, 27, 38, 40, 41, 44, 45, 48, 49, 52, 55, 58, 59, 64, 67, 69 – 74, 78, 79), with consistent evidence found for the effect of alcohol, smoking, and genetics (Table 1). The evidence for the other exposures was limited and/or inconsistent.

### CKDnT causality considerations

Assessments of the causality considerations for each of the exposures are shown in Supplementary Materials: agrochemicals and working in agriculture (S7), heavy metals and hard water (S8), heat stress-dehydration (S9), and other exposures (S10). The study also identified research gaps (S11).

## DISCUSSION

### Agrochemicals

We found consistent evidence for the adverse effect of agrochemicals on chronic kidney disease, and in some studies, an association with end-stage renal failure (see the Supplementary Materials). In our meta-analysis, which included 13 studies from different regional areas, the overall effect was positive, and became significant when cross-sectional studies were removed. It is important to stress that high quality studies found significant associations for specific agrochemicals and in regions not declared as “endemic” (33, 34). These cohort studies not only involved men and women working as applicators and/or mixers of agrochemicals, but also women married to applicators, both of whom live in the same agricultural environment. Among married women who directly applied pesticides, and among women who did not but whose husbands directly applied specific pesticides, the effect observed was positive and also showed a significant biological gradient over time. The effect was also related to cumulative exposure and to having a well for drinking water, a possible source of agrochemical exposure (33, 34).

Although we did not conduct a meta-analysis of specific agrochemicals, there were studies (33, 34) of the agrochemical paraquat that was related both by direct exposure (in men) and indirect exposure (in women married to applicators). A study (26) conducted in Sri Lanka found that drinking water from abandoned wells contaminated with glyphosate and heavy metals increased the risk of CKDnT disease by a factor of 5. In Sri Lanka, few cases of the disease have been reported in an area with similar climatic and agricultural conditions, but where glyphosate was banned during the war years (80). Additionally, another review (81) argues that there is compelling evidence that glyphosate exposure is a significant factor in CKDnT in Mesoamerica.

The adverse effects of pesticides on CKDnT could be further aggravated by the presence of chemicals in the soil and drinking water, with possibly cumulative residual effects on soil and water (80). Comparison of biopsy patterns between endemic and non-endemic regions for those with ESRD due to CKDnT could help to clarify whether it is in fact the same disease (1), as has been done for Sri Lanka and Mesoamerica (3).

In relation to biological plausibility, in their systematic review Valcke and colleagues (5) report experimental and clinical evidence to support the claim that a number of pesticides commonly used in many parts of the world are known human nephrotoxins. They reported an effect on acute kidney injury rather than CKD. Experimental studies in animals describe the effect of glyphosate in the kidney with histological patterns and markers of renal failure similar to CKDnT, including genotoxic and teratogenic effects (80, 82). A recent study also showed kidney damage from a broad-spectrum glyphosate-based herbicide to glyphosate alone (83). In humans, kidney damage due to acute intoxications, including evolution to chronic renal illness, has also been seen (5).

### Metals and hard water

In this review, 23 primary studies of the association between CKDnT and heavy metals and/or hard water were included, mainly from South Asia and non-endemic countries. There was a generally positive association between exposure to heavy metals and CKDnT, particularly for cadmium and possibly lead, and some evidence from cohort studies to support a temporal relationship (see the Supplementary Materials). The studies of cadmium also showed a positive dose-response gradient. The results for the association of arsenic with CKDnT were mixed, but when the dose-response gradient was measured it was mostly positive. There were only two studies of mercury included, and both showed a protective effect.

Heavy metals, such as cadmium, lead, and mercury are established nephrotoxins at high exposure levels (84, 85). Experimental studies reported by a systematic review (18) support the association between arsenic and the development of CKD. The most plausible explanation that was found refers to inflammatory processes and oxidative stress. In one included cohort study the adverse effects of chronic exposure to arsenic were also seen to be reversed with interventions that reduced exposure (22). Some studies have also shown that drinking well water and drinking from abandoned wells—that likely have an even higher concentration of contaminants—in endemic areas increased the risk of CKDnT by 2 and 5 times, respectively (26).

### Heat stress-dehydration

There was no consistent evidence from our systematic review to support the association between CKD and heat stress-dehydration. The studies evaluated assume that changes in osmolarity and other markers in blood and urine provoke repeated episodes of acute renal failure, and from there could lead to the development of CKD. A proposed mechanism by which recurrent or chronic dehydration is capable of causing CKDnT has been put forward by Roncal Jimenez and colleagues (86). It includes increased release of vasopressin, increased oxidative stress by cortical production of uric acid, and hyperuricemia. Meanwhile, two other systematic reviews (one of them of low quality) showed that acute kidney injury is a risk factor for CKD and ESRD in non-agricultural populations and in a hospital environment with known risk factors for CKD (87, 88).

There are reports showing that agricultural workers in Central America are exposed to heat stress (89 – 91) with periods of recurrent dehydration capable of causing alterations of renal function, mainly acute kidney injury, but with no evidence that these conditions can cause CKD (48, 65, 68, 87, 92). Although heat stress and dehydration as causes of chronic kidney damage have some biological plausibility, there are no studies that show indisputably that they are the single or preponderant cause of the onset of the so-called CKDnT. Despite the evidence for the production of chronic kidney damage in animal experiments, there is no solid evidence in humans.

A recent review strongly criticizes the evidence related to heat stress and dehydration as the main cause for CKDnT in Mesoamerica. The authors suggest that, instead, CKDnT is probably a toxic nephropathy (93). Also, the mechanization of agricultural labor, as well as the presence of CKD and ESRD in women who do not work physically (as in Sri Lanka), or in non-tropical regions (such as the United States; 34) go against the heat stress-dehydration hypothesis. Finally, a recent study on the prevalence of early renal damage markers in adolescents with no history of strenuous manual work living in a sugarcane agricultural area with a high number of CKDnT cases would reinforce the role of environmental toxins rather than heat stress-dehydration (94).

### Work in agriculture

This meta-analysis of 15 studies found that working in agriculture significantly increased the risk of having CKD in all regions. The studies included in the qualitative and quantitative synthesis involved nearly 100 000 participants. A dose-response relationship also was found in participants of both sexes who work closer to sea level (compared to highlands) in Central America, with risks for both men and women (44, 57), as well as those who had worked in agriculture for more than 5 years in a non-endemic region (75). Work in agriculture is a wide context exposure that could also include multiple other exposures such as agrochemicals, heavy metals, and/or heat stress-dehydration. It is likely that there is an interaction between these exposures, resulting in varied patterns of damage.

A key strength of this work was the combination of both qualitative and quantitative syntheses, including meta-regression, which ensured that all included studies contributed to the main findings, not just those that reported results as ORs. In addition, by including studies from countries not identified as endemic (e.g., Japan, Mexico, the United States), with similar results, it became apparent that the problem of CKDnT goes beyond “endemic” countries and regions.

#### Limitations.

A study limitation worth noting is the considerable heterogeneity of the included primary studies, which limited our ability to make firm conclusions about the state of the evidence. Also, relevant studies published after August 2017 could not be included (95, 96).

## Conclusions

This meta-analysis showed a statistically significant association between CKD and pesticide use when cross-sectional studies were excluded. Results for heavy metals, hard water, and heat stress were not consistent. All results had substantial heterogeneity. Several other risk factors have been studied, with consistent evidence found for the effects of alcohol, smoking, and genetics; however, some important and complex gaps in knowledge remain.

Based on the existing evidence and the precautionary principle, it is important to implement preventive measures to mitigate the damage caused by CKDnT to agricultural workers and their communities (i.e., improved working conditions, cautious management of agrochemicals, etc.). More high-quality research is needed to measure impact and to build the evidence base.

## Author contributions.

EC, LR, MH, and VE wrote the protocol. EC, MH, and EI assessed the main exposures against the Bradford Hill considerations. EC, LR, and VE did the searches. EC and MH selected studies for inclusion. EC, MH, EI, and JSV assessed the methodological quality of studies and extracted data. MH did the meta-analysis and meta-regression. All authors reviewed and approved the final version**.**

## Funding.

This review was funded by PAHO/WHO. The views expressed in this article are those of the authors and do not necessarily represent those of PAHO/WHO. The funders had no role in the study design, data collection or analysis, decision to publish, or preparation of the manuscript.

## Disclaimer.

Authors hold sole responsibility for the views expressed in the manuscript, which may not necessarily reflect the opinion or policy of the RPSP/PAJPH or the Pan American Health Organization (PAHO).
